# Advances in New Physical Layer Technologies for Next-Generation Wireless Communications

**DOI:** 10.3390/e27060616

**Published:** 2025-06-10

**Authors:** Lei Liu

**Affiliations:** College of Information Science and Electronic Engineering, Zhejiang University, Hangzhou 310027, China; lei_liu@zju.edu.cn

## 1. Introduction

The world of wireless communication is undergoing an exhilarating transformation. As applications advance at an unprecedented pace, communication services have diversified, and deployment environments have grown increasingly complex—ushering in both extraordinary opportunities and formidable challenges for next-generation systems. As the cornerstone of wireless systems, physical layer technologies are designed to combat common impairments in wireless channels—such as fading, delay spread, Doppler shifts, interference, and noise—ensuring reliable and stable data transmission. This, in turn, supports the seamless operation of upper-layer services. To address the escalating demands of next-generation networks, the evolution of physical layer technologies is advancing across several key frontiers, including, but not limited to, the following:

- *Multicarrier Modulation:* Multicarrier modulation is highly effective in enhancing spectral efficiency and mitigating the adverse effects of complex wireless channels. Multicarrier modulation has evolved from traditional orthogonal frequency division multiplexing (OFDM) to more advanced schemes such as orthogonal time frequency space (OTFS) [1], affine frequency division multiplexing (AFDM) [2], and interleaved frequency division multiplexing (IFDM) [3], in order to meet the demands of high-mobility communication. While OFDM offers simplicity and high efficiency in static environments, its performance degrades significantly in time-varying scenarios. OTFS and AFDM achieve diversity gains by separating signals in the delay-Doppler domain but suffer from a lack of efficient detection algorithms. IFDM constructs a dense equivalent channel matrix to ensure statistically stationary transmission of signals. These modulation techniques serve as a cornerstone for enabling high-mobility communication technologies.
- *Channel Estimation and Signal Detection:* Channel estimation and signal detection are essential components of the receiver for ensuring reliable wireless communications, particularly in complex and high-dimensional systems. While classical algorithms like least squares (LS) and minimum mean square error (MMSE) offer baseline performance, they fall short under massive multiple input multiple output (MIMO), low SNR, or non-linear hardware constraints. Recently, approximate message passing (AMP)-based algorithms and their variants—such as orthogonal AMP (OAMP) [4], vector AMP (VAMP) [5], and memory AMP (MAMP) [6]—have emerged as powerful tools for large-scale signal detection. These algorithms exploit the statistical structure of channels and signals to achieve near-optimal performance with low complexity and provable convergence in certain conditions. However, challenges remain in extending AMP to correlated priors, finite-length effects, and ensuring robustness in dynamic or imperfect channel state information (CSI) scenarios. Integrating AMP with learning-based or model-driven frameworks is a promising direction for next-generation communication systems.
- *Channel Coding and Decoding Design*: With the widespread adoption of the Internet of Things (IoT) and the growing number of wireless device connections, many communication services are placing new demands on low latency, high reliability, and short-packet transmission. These requirements drive the need to revisit channel coding and decoding design under finite-length and multi-user communication constraints. However, unlike long-length codes, finite-length codes still lack a unified design framework, practical tools, and low-complexity, efficient decoding algorithms. Furthermore, most traditional channel coding schemes are tailored for point-to-point channels, making them inadequate for effectively managing interference in multi-user scenarios [7]. Additionally, the development of flexible, variable-rate coding schemes is also essential for the practical deployment of channel coding in real-world systems.
- *Information Theory Evolution:* As practical communication scenarios become increasingly complex, classical information theory—rooted in idealized or simplified assumptions—can no longer accurately characterize the performance limits of emerging systems such as the constrained (e.g., peak-limited and/or band-limited) channel, millimeter-wave, terahertz, ultra-large-scale MIMO, near-field communication, and semantic communication. Consequently, it is essential to develop modern information theory that accounts for practical communication constraints [8]. This modern theory serves as both an evolution and a complement to classical information theory, providing a more accurate theoretical foundation for the design and performance analysis of next-generation communication systems.
- *Integrated Sensing and Communication (ISAC)*: ISAC has emerged as a key enabling technology for next-generation wireless networks, aiming to unify the traditionally separate functions of wireless sensing and data communication [9]. Recent advances in ISAC have focused on joint waveform design, resource allocation, and signal processing algorithms that enable a single hardware and spectral resource to serve both functions efficiently. Cutting-edge research has proposed dual-functional waveforms based on OFDM, OTFS, AFDM, and other modulation schemes, which offer high spectral efficiency and robustness in dynamic environments. ISAC systems have been extended to support large-scale MIMO, near-field communication, and high-mobility scenarios, demonstrating significant improvements in both sensing accuracy and communication reliability. Additionally, theoretical frameworks for analyzing the rate-sensing trade-off and performance bounds are being developed to guide practical system design. Overall, ISAC represents a paradigm shift in wireless system architecture, promising reduced hardware costs, enhanced spectral efficiency, and improved environmental awareness, and is expected to play a central role in future intelligent and ubiquitous wireless infrastructures.
- *Reconfigurable Intelligent Surface (RIS)*: RIS is a key enabler for future 6G networks, capable of reconfiguring the wireless environment to enhance coverage, spectral efficiency, and energy efficiency [10]. By intelligently controlling wave reflection through nearly passive elements, RIS reduces hardware costs and power consumption. Recent progress includes joint beamforming, channel estimation, and integration with MIMO, THz, and ISAC systems. Future research should focus on scalable deployment strategies, real-time control under dynamic environments, and unified theoretical frameworks to guide practical RIS system design.
- *Artificial Intelligence for Radio Access Networks (AI-RAN)*: AI-RAN represents a transformative paradigm that deeply integrates AI technologies [11]—such as machine learning and deep learning—into the design, optimization, and operation of wireless access networks. By leveraging data-driven models, AI-RAN enables intelligent resource allocation, real-time traffic prediction, adaptive interference management, and proactive fault detection, thereby enhancing the efficiency, flexibility, and reliability of wireless systems. It plays a pivotal role in supporting diverse 5G and future 6G applications, including autonomous driving, industrial IoT, and immersive media. However, AI-RAN still faces several key challenges: ensuring the generalization and robustness of learning models under dynamic and complex wireless environments, achieving low-latency and energy-efficient AI inference at the network edge, and establishing trustworthy and interpretable AI frameworks. Addressing these challenges is critical to fully realizing the potential of AI-RAN in next-generation wireless networks.
- *Communication Technology-Driven Low-Altitude Economy:* Physical layer communication technologies are crucial for the low-altitude economy, enabling reliable and efficient links in environments with high mobility and dynamic channels [12], such as low earth orbit (LEO) satellites and unmanned aerial vehicle (UAV) networks. Key advances include robust modulation and coding schemes (e.g., OTFS, IFDM), spatial techniques like MIMO and IRS, and the use of high-frequency bands for enhanced capacity. Challenges remain in handling rapidly varying channels, interference management, low-power receiver design, and ensuring security under hardware constraints. Overcoming these issues is essential for the continued growth of low-altitude communication systems.

In summary, this Special Issue is organized to provide a focused platform for presenting and discussing the latest advances in physical layer communication technologies. It aims to gather high-quality contributions that address emerging challenges and explore innovative solutions in areas such as channel coding, modulation, channel estimation, detection algorithms, and integrated sensing and communication.

Specifically, in system capacity analysis under practical constraints, a tight lower bound is derived for peak-power-limited (PPL) additive white Gaussian noise (AWGN) channels using a geometry-based approach [13], while a constrained Markov decision process (CMDP)-based strategy improves user-perceived capacity through joint power-rate adaptation [14]. In channel coding and decoding, efforts focus on reducing latency and complexity: entropy-aware layered scheduling enhances low-density parity-check (LDPC) decoding [15], and hybrid window decoding lowers the complexity of joint source–channel anytime codes [16]. Additionally, analog fountain code designs [17] and low-density algebra-check (LDAC) optimization via extrinsic information transfer (EXIT) charts improve coding efficiency in short-block and low-rate scenarios [18]. On modulation waveforms, chirp-BOK modulation is introduced for tropospheric scattering in deep fading environments [19], a generalized filter bank OFDM scheme enhances bandwidth efficiency for 6G THz systems [20], and random frequency division multiplexing (RFDM) integrates compressed sensing and deep neural networks for robust detection in dynamic channels [21]. Emerging technologies are also explored for their potential to jointly improve transmission efficiency. For example, refs. [22,23] propose optimal decoding order and resource allocation strategies for non-orthogonal multiple access (NOMA) and NOMA-based satellite–terrestrial networks. In reconfigurable intelligent surface (RIS)-assisted systems, ref. [24] presents a simultaneously transmitting and RIS (STAR-RIS)-aided cooperative rate splitting (CRS) framework with optimized beamforming and rate allocation, while ref. [25] proposes a three-step intelligent reflecting surface (IRS) deployment strategy tailored for coal mine tunnels. A comprehensive tutorial on near-field integrated sensing and communication (ISAC) [26] highlights the benefits of jointly optimizing sensing and communication using shared RF resources. Furthermore, recent articles tackle advanced challenges including high-speed railway [27] and UAV terahertz channel modeling [28], quantum entanglement robustness [29], deep learning-based service function chain (SFC) reconfiguration for the 6G IoT scenario [30], and secure federated learning under ultra-reliable and low-latency communication (URLLC) [31].

In summary, the aforementioned studies offer innovative models, algorithms, and insights at the physical layer, providing valuable guidance for future research.

## 2. An Overview of Published Articles

In the study of system capacity analysis, the first article [13] explores peak-power-limited AWGN channels, using a geometry-based approach to provide tighter lower bounds on channel capacity. These bounds improve power efficiency by factors of 3.3 and 8.6, with a 6 dB penalty at high SNRs, offering insights for modulation and coding schemes. The second article [14] studies user-perceived throughput in mobile multimedia services, showing that capacity is limited with only channel state information at the receiver (CSIR), but improves significantly with both CSIR and channel state information at the transmitter through power and rate adaptation. They propose a CMDP-based approach, revealing that the optimal policy follows a threshold rule.

In the study of channel coding and decoding, the third article [15] tackles high decoding latency in LDPC codes, proposing two techniques (serial entropy feature-based layered normalized min-sum and parallel entropy feature-based layered normalized min-sum) to reduce latency by dynamically allocating layered processing units based on bit reliability. Experimental results show significant efficiency improvements. The fourth article [16] reduces decoding complexity in joint source–channel anytime coding (JSCAC) systems using the hybrid window decoding (JHWD) technique, introducing an adaptive decoding structure and an improved density evolution algorithm. The JHWD algorithm is proven to be efficient for low-complexity decoding in JSCAC and similar systems. The fifth article [17] proposes an improved progressive edge-growth construction for analog fountain codes (AFCs), allocating weight coefficients in descending order. Simulation results show it achieves a low block error rate (BLER) in short block lengths. The sixth article [18] explores the use of EXIT charts to optimize LDAC codes and construct a low-rate LDAC code. They also establish an iterative decoding model and demonstrate a low-rate LDAC code with enhanced coding gain through simulations.

In the study of designing modulation waveforms, the seventh article [19] studies chirp-BOK (binary orthogonal keying) modulation in troposphere scattering systems, demonstrating significant BER performance improvements with the optimal configuration. The eighth article [20] introduces the generalized filter bank OFDM waveform for THz communication in 6G networks, improving bandwidth efficiency and reducing complexity compared to cyclic prefix OFDM (CP-OFDM). The ninth article [21] presents an RFDM method for multicarrier modulation in mobile time-varying channels, utilizing compressed sensing (CS) and deep neural networks (DNNs) for signal detection. Simulation results show that the proposed method significantly improves bit error rate (BER) performance, offering a promising approach for enhancing spectrum efficiency in multi-subcarrier, multi-antenna, and multi-user systems.

In the study of technologies for improving transmission efficiency, the tenth article [22] addresses the optimal decoding order and power allocation for sum throughput maximization in downlink NOMA systems over Nakagami-m channels, deriving a closed-form expression for outage probability, analyzing its behavior in high-SNR conditions, and proving the optimal power allocation range for each decoding order. A joint optimization problem for decoding order and power allocation is formulated and solved through an efficient search, validated by Monte Carlo simulations. The eleventh article [23] investigates a NOMA-based integrated satellite–terrestrial network, proposing a two-stage algorithm for resource optimization, with simulation results showing its effectiveness. The twelfth article [24] proposes a STAR-RIS-assisted cooperative rate splitting framework with six transmission modes to maximize the minimum user rate. They introduce a successive convex approximation (SCA)-based alternative optimization (AO) algorithm for joint optimization and a low-complexity algorithm for passive beamforming. Simulation results show significant improvements in user fairness and performance, with the low-complexity method achieving near-AO results at lower computational cost. The thirteenth article [25] investigates a three-step joint rate optimization method for IRS-assisted wireless communication in coal mines. It optimizes the IRS deployment position, transmit power, and phase shifts to maximize achievable rates. To solve the non-convexity of the optimization problem for the IRS deployment position, logarithmic operations, Taylor relaxation, and two auxiliary variables were introduced to transform it into a convex problem. Simulation results show improvements of 12.32% to 54.17% in achievable rates compared to other schemes. The IRS deployment position converges to an optimal location regardless of its initial placement. This paper also examines the impact of tunnel wall roughness, demonstrating superior performance in coal mine scenarios. The fourteenth article [26] provides an overview and tutorial on near-field (NF) ISAC technology, which offers significant advantages over traditional far-field (FF) ISAC systems by enabling simultaneous communication and sensing using shared RF resources. This paper analyzes NF and FF systems, explores their applicability in various scenarios, and discusses different channel models. It also highlights the benefits of ISAC, examines narrow-band and wide-band NF-ISAC systems, and presents case studies and simulations. In addition, it reviews current research, key methodologies, challenges, and future research directions for NF-ISAC.

In the study of complex communication scenarios, the fifteenth article [27] addresses coverage analysis for high-speed railway (HSR) communications, proposing a channel model incorporating path loss, shadowing, and small-scale fading. It derives expressions for edge coverage probability and cell coverage area, validating the results with Monte Carlo simulations. The sixteenth article [28] proposes a 3D time-varying channel model for UAV-based dual-mobility systems in the THz band, considering scattering, reflection, and atmospheric absorption, with simulation results verifying its accuracy. The seventeenth article [29] investigates the entanglement robustness of Dicke–W and GHz mixed states under varying mixing ratios. Simulation results demonstrate that the entanglement robustness increases when the probability ratio of Dicke and W states is greater than or equal to 3/2, while it follows a different trend for ratios below 3/2. The eighteenth article [30] applies deep learning to service function chain reconfiguration to meet the highly dynamic service requirements in the future 6G Internet of Things (IoT) scenario, introducing the knowledge-assisted actor–critic (AC) proximal policy optimization (KA-ACPPO)-based algorithm, which improves flexibility and reduces computing costs while ensuring optimal quality of service (QoS). The nineteenth article [31] discusses physical layer security in wireless hierarchical federated learning (WHFL), proposing a secure finite block-length approach for multi-antenna URLLC-WHFL systems, which provides near-perfect secrecy without compromising learning performance.

## 3. Conclusions

This Special Issue highlights the pivotal role of physical layer technologies in addressing the evolving demands of modern and future wireless communication systems. Through diverse contributions spanning capacity analysis, coding and decoding, waveform design, and emerging technologies such as NOMA, RIS, ISAC, and THz communications, the featured works collectively advance our understanding of robust, efficient, and adaptive physical layer solutions. By tackling both fundamental challenges and application-driven scenarios, these studies lay a solid foundation for future innovations in wireless communication, offering practical insights and theoretical tools to guide the development of next-generation networks.
